# Pilot-scale production and characterization of liquid fuel from mixed non-recyclable plastics for potential steam-generation applications

**DOI:** 10.1039/d6ra06450g

**Published:** 2026-09-16

**Authors:** Kanokwan Panmak, Yuvarat Ngernyen, Supattra Budsaereechai

**Affiliations:** a Department of Chemical Engineering, Faculty of Engineering, Khon Kaen University Khon Kaen 40002 Thailand; b Department of Mechanical Engineering, Faculty of Arts and Science, Chaiyaphum Rajabhat University 36000 Thailand pla.supattra.cpru@gmail.com

## Abstract

Post-sorting non-recyclable plastic residues pose significant management challenges due to the limitations of mechanical recycling. This study demonstrates a novel, direct vacuum-assisted pilot-scale batch pyrolysis of mixed plastic rejects dominated by polypropylene (PP, ≥45 wt%), low-density polyethylene (LDPE, ≥45 wt%), polystyrene (PS, ≤5 wt%), and high-density polyethylene (HDPE, ≤5 wt%) in an insulated 1,000 L reactor without continuous inert-gas purging. Under optimized operating conditions (30 min air pre-evacuation at 15–20 kPa, 20 kg feed loading, LPG heat input at ∼25% maximum flow, and 1 h reaction at ∼450 ^°^C), a high condensable liquid yield of ∼80 wt% was achieved. Comprehensive structural and compositional characterization *via* FTIR confirmed the pyrolytic liquid's aliphatic-rich nature, featuring strong C–H stretching vibrations (2956–2850 cm^−1^), C–H bending (1455 cm^−1^ and 1375 cm^−1^), and unsaturated alkene C=C bonds (1640 cm^−1^ and 990–890 cm^−1^). GC-MS analysis further revealed a carbon number distribution highly compatible with commercial heavy fuel oil, dominated by heavy (>C_13_, 63.02% area) and middle-distillate (C_10_–C_13_, 23.28% area) fractions, with low total aromaticity (11.57% area in >C_13_). Fuel-property benchmarking demonstrated a high heating value (43 115–43 500 cal g^−1^) and kinematic viscosity at 50 °C (∼12 cSt) closely matching commercial furnace oil (43 619 cal g^−1^ and 12 cSt), alongside environmentally advantageous ultra-low sulfur (0.0038–0.0057 wt%) and ash contents (<0.001 wt%). Washing pretreatment offered no improvement in liquid recovery while significantly increasing water and sediment content (20 vol% *vs.* 0.9 vol% for unwashed feed) due to trapped residual moisture. Distance-resolved air-quality monitoring confirmed rapid dispersion of particulate matter (PM_2.5_: 293 → 165 → 22 µg m^−3^; PM_10_: 384 → 134 → 28 µg m^−3^ at 0/5/10 m), establishing a safe operational buffer distance of ≥10 m. Overall, this single-step, direct vacuum-assisted process offers an energy-efficient, low-cost solution for converting municipal plastic waste into high-quality commercial fuel oil substitutes without requiring catalytic upgrading or post-pyrolysis distillation.

## Abstract 2

Post-sorting non-recyclable plastic residues pose significant management challenges due to the limitations of mechanical recycling.

## Graphical abstract

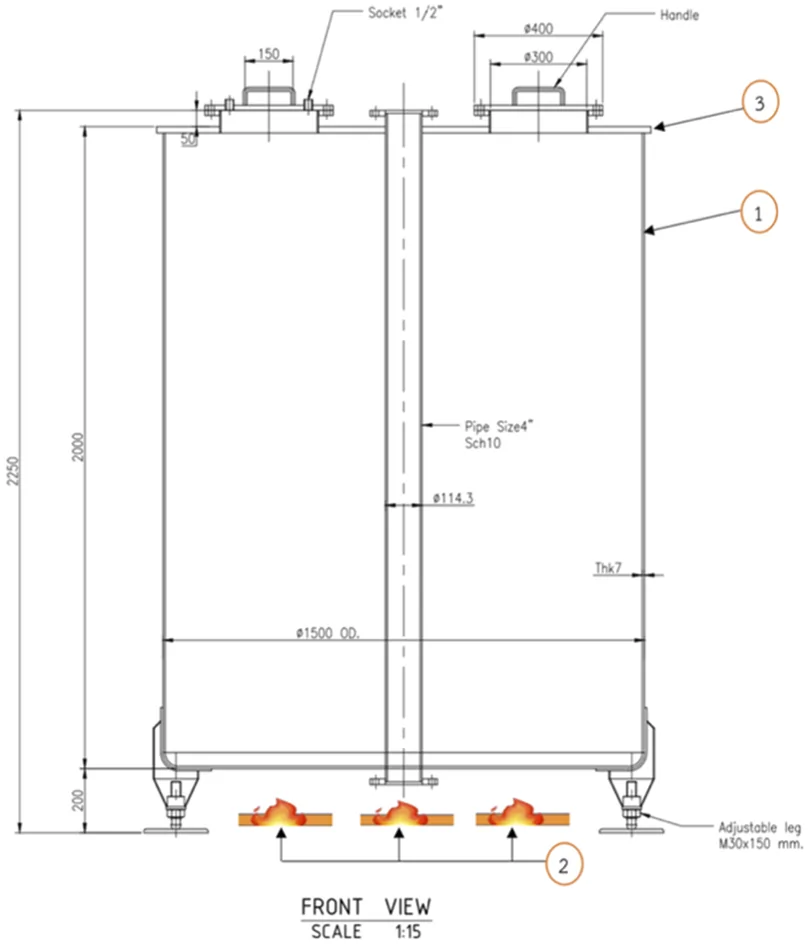

## 1 Introduction

Plastic is widely used in daily life, particularly as packaging for food, beverages, and consumer products, due to its lightweight, durable, and inexpensive nature.^1^ However, plastic waste persists in the environment for extended periods and contributes to environmental pollution when improperly managed. Because packaging dominates the post-consumer plastic waste stream, sorting residues are often enriched in films, flexible polyolefins, and multilayer packaging that are difficult to recycle mechanically even after separation. Although mechanical recycling can recover a portion of the plastic waste stream, it requires clean and well-sorted plastics.^1–3^ In practice, recycling facilities generate significant amounts of post-sorting residues that cannot be recycled due to contamination, mixing, or the presence of problematic materials.^4–7^ These non-recyclable residues are often stockpiled, landfilled, or improperly burned, creating local environmental^8^ and health concerns^9^ and placing additional pressure on municipal waste management systems.^10^ This limitation provides a strong rationale for conversion routes that can tolerate mixed and partially contaminated feedstocks rather than requiring near-virgin material.

Converting non-recyclable plastic residues into usable energy is an attractive option, especially in regions with significant industrial heat demand.^11^ Pyrolysis is a thermochemical process that thermally decomposes polymers under oxygen-limited conditions to produce condensable hydrocarbons (oil/wax), non-condensable gases, and a solid residue.^12–14^ While many studies have reported plastic pyrolysis at the laboratory scale, large-scale deployment requires evidence under more practical conditions, including larger batch operations,^15–17^ feed variability,^18,19^ simple oxygen control strategies that do not rely on continuous inert-gas purging, and assessments of air quality^18^ around the unit during operation.^10^ For deployment beyond the laboratory, operational simplicity, batch capacity, feed heterogeneity, and local emission behavior are as important as product yield, because these factors determine whether the system can be operated economically and safely in real waste-management settings.

Although several studies have investigated the conversion of mixed plastic waste into liquid fuels, most research remains confined to bench-scale setups or relies on multi-stage refining processes. For instance,^20^ demonstrated a pilot-scale approach to synthesize standard diesel from mixed plastics *via* thermal pyrolysis at 540 °C; however, their framework required a secondary vacuum distillation step (170–380 °C) to isolate a refined diesel cut (52.5–53.0 wt%). Such multi-step configurations inevitably increase capital investment, operational complexity, and overall process energy intensity.^21,22^

To address these limitations, the present study introduces a novel, single-step pilot-scale batch pyrolysis strategy operating at a lower thermal severity with an enhanced feed capacity of up to 40 kg. The key innovation lies in implementing a pre-heating vacuum evacuation stage, which effectively suppresses oxidative side reactions and enhances the thermal cracking efficiency of contaminated mixed plastic rejects. By bypassing post-pyrolysis distillation, this direct vacuum-assisted process offers an efficient, low-energy, and cost-effective pathway for municipal-scale plastic waste valorization without compromising fuel yield or quality.

In this work, mixed non-recyclable plastic residues dominated by PS, PP, HDPE, and LDPE^23^ were processed in an insulated pilot-scale batch reactor with a practical feed capacity of at least 1000 liters per batch. These polymers are common in post-sorting packaging residues and represent major hydrocarbon-forming fractions for liquid-oriented pyrolysis air was evacuated using a vacuum pump before heating to limit oxygen and reduce operational complexity. Key operating parameters,^24^ vacuum time, production time, feed loading, and LPG heat input, were varied to determine the operating window that maximizes the yield of a fuel-oil-like condensed liquid. The necessity of washing pretreatment was also evaluated to determine whether preprocessing can be simplified without compromising performance.^25^

To support the application as a substitute for commercial fuel oil used for steam generation, the condensed liquid was benchmarked against a reference fuel oil using standard fuel properties. In this study, the target application is boiler/steam-service substitution rather than transportation use; therefore, benchmarking against commercial fuel oil is more relevant than comparison with highly upgraded refinery-grade fuels. Additionally, air quality indicators and exhaust gas parameters were measured at different distances from the product storage area, providing practical safety-relevant information for pilot and community deployment. Together, these results aim to establish not only a high-yield operating condition but also an evidence-based pathway for translating plastic waste pyrolysis into a deployable, community-scale waste-to-fuel option.

## 2 Experimental

### 2.1 Materials and methods

#### 2.1.1 Feedstock preparation and pretreatment

Mixed non-recyclable plastic residues were collected from post-sorting rejects at a municipal waste management facility in Chaiyaphum, Thailand. The raw feed was manually sorted to retain four dominant target polymers: polypropylene (PP, ≥45 wt%), low-density polyethylene (LDPE, ≥45 wt%), polystyrene (PS, ≤5 wt%), and high-density polyethylene (HDPE, ≤5 wt%). Non-target materials and foreign contaminants were removed to prevent reactor clogging and incomplete thermal degradation. The sorted plastic mixture was sun-dried for 7 days, followed by hot-air drying to ensure low baseline moisture.

To evaluate the necessity of feed washing, comparative trials were performed using unwashed plastics alongside samples subjected to 1–3 washing cycles with tap water. Washed samples were air-dried for either 5 or 7 days prior to pyrolysis. Moisture content before and after drying was measured in accordance with ASTM D3173.

#### 2.1.2. Pilot-scale pyrolysis system and operation

**Fig. 1:**
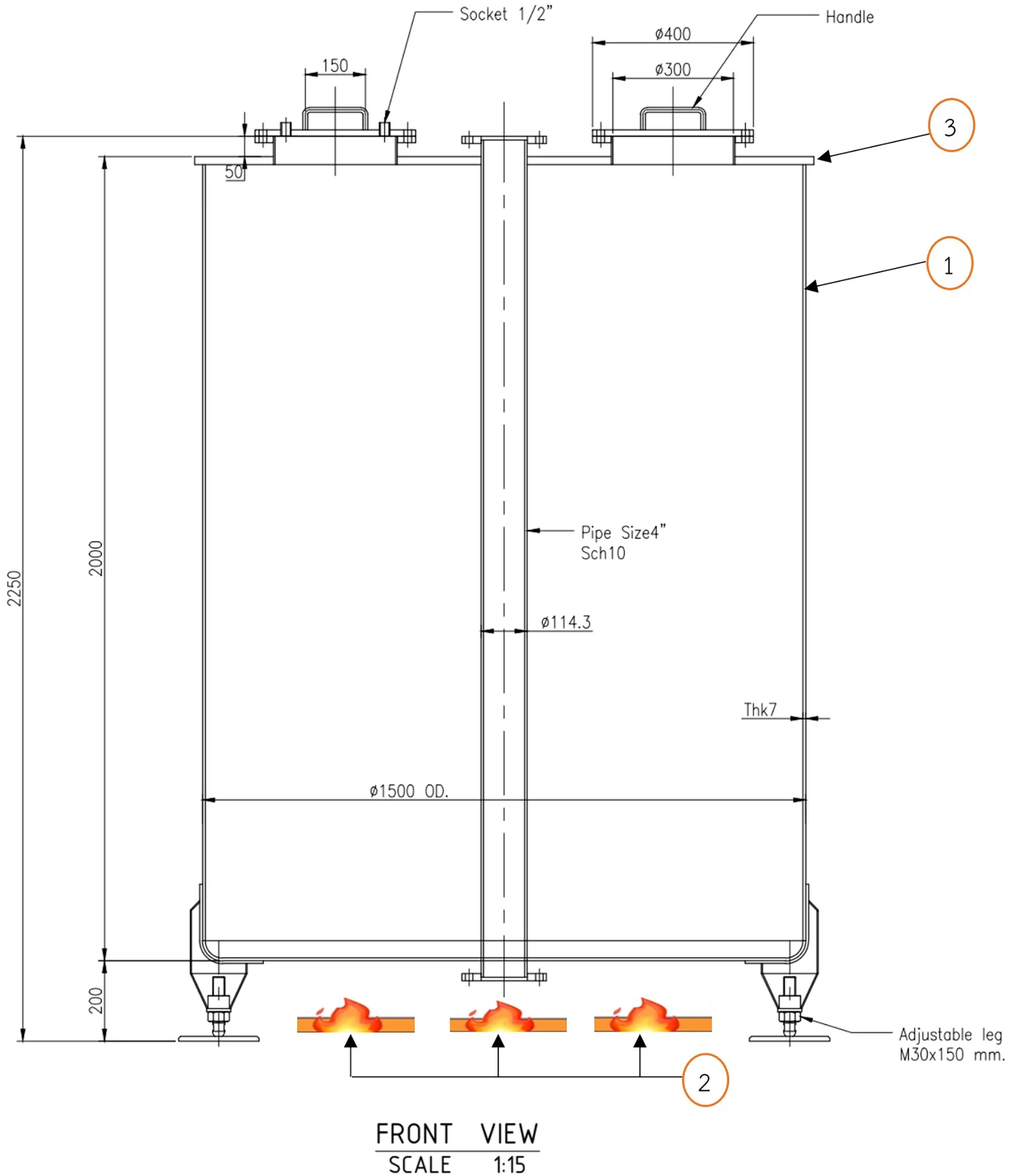
Pilot-scale batch pyrolysis system for mixed plastic waste conversion.

Pyrolysis experiments were performed in a pilot-scale, thermally insulated batch reactor with a working volume of approximately 1000 L (Fig. 1). The system temperature profile was monitored using three calibrated K-type thermocouples positioned at the outer bottom surface, the core liquid/melt region, and the upper gas-phase zone. Heat was supplied by liquefied petroleum gas (LPG) burners, maintaining a constant heating rate of 10 °C min^−1^. The maximum LPG consumption rate corresponded to 20 kg h^−1^ at 100% valve opening.

Prior to thermal ramping, atmospheric air was evacuated from the sealed reactor using a vacuum pump (capacity ≥140 L min^−1^) for 0 to 60 min to establish a low-oxygen environment (<1.0 vol% O_2_, corresponding to an absolute pressure of 15–20 kPa), eliminating the requirement for continuous inert-gas purging. Condensable vapors generated during pyrolysis were passed through a water-cooled condensation unit and collected in sealed 200-L steel receiver tanks. Operational parameters—including reactor temperature, reaction time, and collected product mass—were automatically logged at 5-min intervals using a data acquisition system.

#### 2.1.3. Experimental design and process optimization

To enhance liquid fuel yields while minimizing operational expenditures (OPEX), pre-reaction air evacuation (0, 30, and 60 min) was evaluated as a cost-effective alternative to continuous nitrogen purging. Pre-evacuation was conducted using a vacuum pump with a displacement capacity of ≥140 L min^−1^. For initial screening, 5 kg of prepared plastic waste feedstock was loaded into the reactor. The operational cycle comprised a 1-h thermal melting phase followed by a 1-h pyrolysis reaction phase. Thermal energy input was regulated by throttling the liquefied petroleum gas (LPG) control valve to a 1/4 open position (25% maximum flow rate). System temperature, elapsed time, and instantaneous product yields were monitored at 5-min intervals.

The effect of initial feedstock loading capacity (5, 10, 20, 30, and 40 kg per batch) was systematically investigated. The prepared plastic waste was charged into the pilot-scale reactor and thermally cracked under an inert, oxygen-free atmosphere following a 30-min pre-evacuation step (15–20 kPa). The LPG fuel supply was maintained at a 25% valve opening (corresponding to a maximum rating of 20 kg h^−1^ total consumption). Operational parameters were recorded every 5 min throughout the sequential 1-h melting and 1-h pyrolysis phases.

Thermal severity was varied by adjusting the LPG fuel supply valve to 1/10, 1/4, 3/10, 4/10, and 1/2 open positions, corresponding to 10%, 25%, 30%, 40%, and 50% of maximum thermal input capacity, respectively. The feedstock mass was held constant at 10 kg per batch, maintaining identical 30-min evacuation, 1-h melting, and 1-h reaction protocols.

To evaluate feedstock pretreatment effects, unwashed plastic waste was benchmarked against washed feeds: single-washed samples dried for 5 and 7 days, and samples washed 2 and 3 times with a fixed 7-day drying period. Feed loading (10 kg per batch), evacuation time (30 min), LPG input (25%), and heating cycles (1-h melting +1-h pyrolysis) were kept uniform across all treatments.

#### 2.1.4 Product mass balance and liquid yield determination

Liquid yield (*Y*_liquid_, wt%) was determined gravimetrically using eqn (1):1
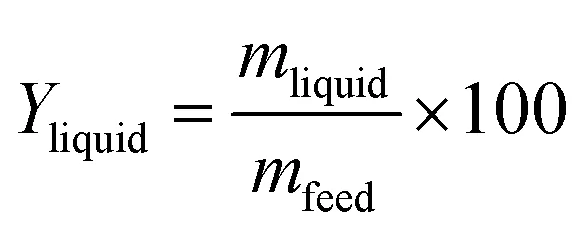
where *m*_liquid_ is the mass of condensed liquid and *m*_feed_ is the initial plastic mass. Solid char residue (*m*_char_) was quantified post-run by direct weighing of the reactor cavity. The non-condensable gaseous fraction (*Y*_gas_, wt%) was derived *via* mass conservation:*Y*_gas_ = 100 − (*Y*_liquid_ + *Y*_solid_)

#### 2.1.5. Product characterization

Physical and fuel properties of the pyrolytic liquid were benchmarked against commercial heavy fuel oil (supplied by Cargill Meats Thailand Co., Ltd) following standard procedures:

• Fuel properties: total sulfur content (ASTM D5453-24), specific gravity at 15.6 °C (ASTM D4052-22), kinematic viscosity at 50 °C (ASTM D445-24), flash point (ASTM D93-20), pour point (ASTM D97-17b), higher heating value (HHV; Gallenkamp Auto Bomb Calorimeter), ash content (ASTM D482-19), and water/sediment content (ASTM D1796-11).

• Hydrocarbon group composition: quantified according to ASTM D6730-22 for paraffins, naphthenes, olefins, aromatics, and >C_13_ heavy fractions.

• FTIR spectroscopy: functional groups were identified using a Fourier Transform Infrared Spectrometer (Tensor 27, Bruker, Germany) over 4000–−400 cm^−1^ at a spectral resolution of 4 cm^−1^.

• GC-MS analysis: molecular composition was determined using a gas chromatograph-mass spectrometer (Clarus SQ 8T, PerkinElmer, USA) equipped with a Clarus 680 GC and an Elite-5MS capillary column (30 m length × 0.25 mm internal diameter × 0.25 µm film thickness). Ultra-pure helium (99.999%) served as the carrier gas at a constant flow rate of 1.0 mL min^−1^. Liquid samples (1 µL) were injected in split mode (1 : 50) at an injector temperature of 280 °C. The GC oven temperature was programmed from 40 °C (2-min hold) to 280 °C at 10 °C min^−1^, with a final hold of 10 min. Electron ionization (EI) was operated at 70 eV with an ion source temperature of 230 °C and a transfer line temperature of 280 °C. Mass spectra were acquired over an *m*/*z* range of 35–−500 amu. Compounds were identified by matching with the NIST 17 Mass Spectral Library (similarity index >80%), and relative abundances were quantified *via* total chromatographic peak area normalization (area%).

#### 2.1.6. Operational air quality and emission monitoring

Ambient air pollutants and exhaust gases were monitored during steady-state liquid generation (0.5–1 h after initiation) at radial distances of 0, 5, and 10 m from the product storage area. Tests were conducted under controlled field conditions with ambient temperatures between 33 and 38 °C. Ambient concentrations of formaldehyde (HCHO), total volatile organic compounds (TVOC), particulate matter (PM_2.5_ and PM_10_), carbon monoxide (CO), and carbon dioxide (CO_2_) were measured using a calibrated MT26co air-quality monitor. Exhaust gas parameters (NO_*x*_, O_2_, CO, and CO_2_) were measured simultaneously using a Model 5002 Exhaust Gas Analyzer.

## 3 Results and discussion

### 3.1. Effect of operating parameters on liquid yield

**Fig. 2:**
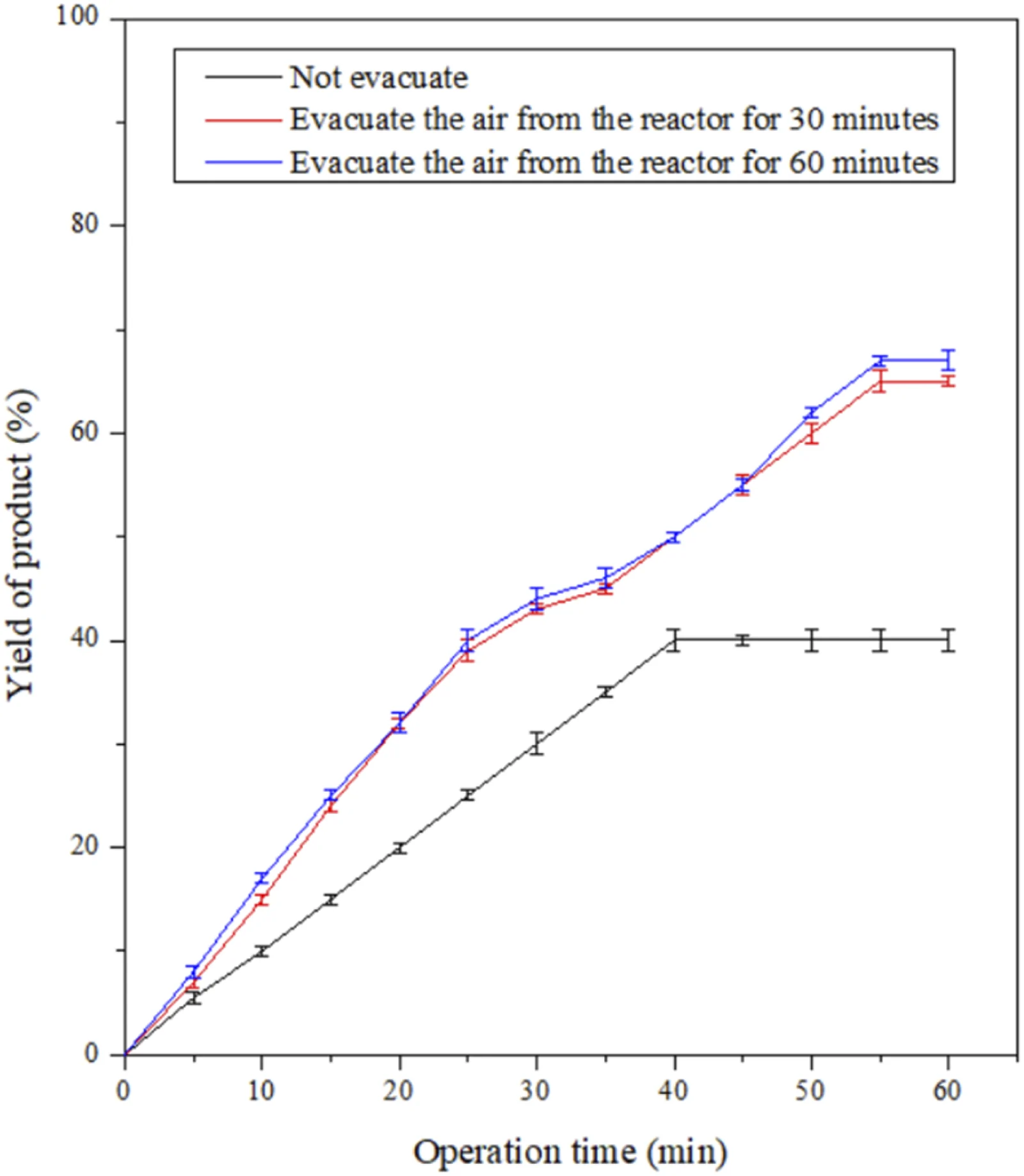
Effect of air evacuation (vacuum) time on liquid yield.

Liquid yield was strongly governed by air evacuation duration, feedstock loading, and LPG heat input. Extending the pre-evacuation time from 0 to 30 min markedly elevated the liquid yield from ∼40 wt% to ∼67 wt%. However, further extension to 60 min provided only a negligible gain, establishing 30 min as the practically sufficient threshold (Fig. 2). These results indicate that depressing the residual oxygen concentration suppressed undesirable side-reactions and enhanced vapor condensation. Pre-evacuation for 30 min efficiently purged atmospheric air from the reactor, reducing residual oxygen to <1.0 vol%, corresponding to an absolute pressure of 15–20 kPa. Maintaining a strictly inert, oxygen-free atmosphere is essential to prevent partial oxidative degradation, which otherwise promotes the formation of oxygenated hydrocarbon by-products (*e.g.*, aldehydes, ketones, carboxylic acids, and phenols).^26,27^ Suppressing these oxygenates enhances the higher heating value (HHV) as well as the long-term chemical and thermal stability of the pyrolytic oil, thereby mitigating sludge formation and acidity-induced corrosion.^28–30^

**Fig. 3:**
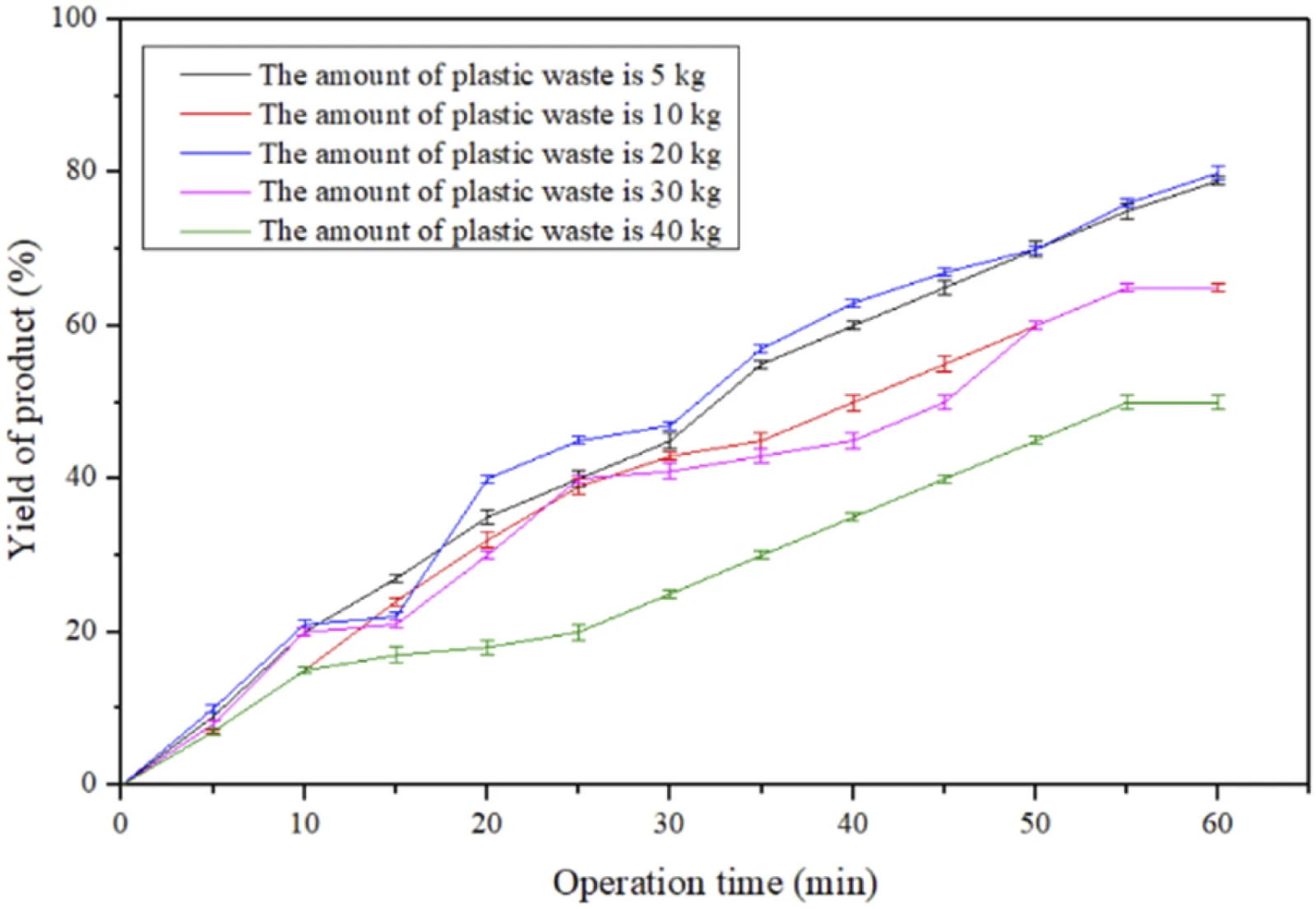
Effect of feed loading on liquid yield in pilot-scale batch operation.

Feedstock loading also exerted a profound effect on liquid recovery. Liquid yield increased as feed mass was raised up to 20 kg, but progressively declined at 30–40 kg, dropping to ∼50 wt% at 40 kg (Fig. 3). This yield reduction stems from heat transfer limitations inherent to pilot-scale batch operations, including uneven thermal gradients, melt accumulation, and restricted vapor flow through a thicker plastic bed.

**Fig. 4:**
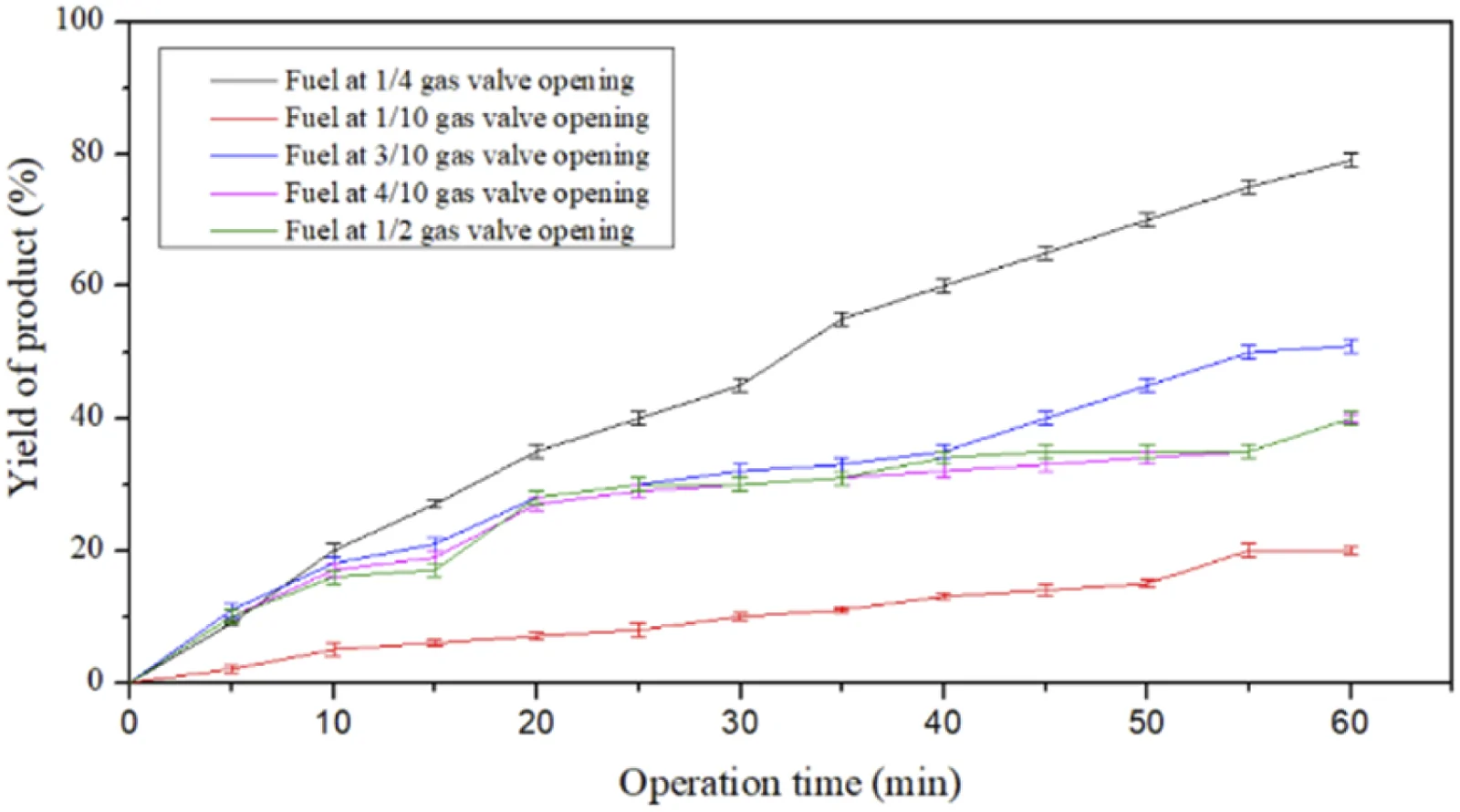
Liquid yield (%) *versus* production time at 10, 25, 30, 40, and 50% of the maximum LPG fuel flow rate (where the maximum flow rate corresponds to a total LPG consumption of 20 kg h^−1^ per experiment).

LPG heat input displayed a similar trend. Optimal liquid yield was attained at ∼25% of the maximum LPG flow setting (Fig. 4). At higher LPG inputs, excessive thermal severity likely promoted secondary thermal cracking, thereby converting condensable vapors into lighter, non-condensable gaseous hydrocarbons.

**Fig. 5:**
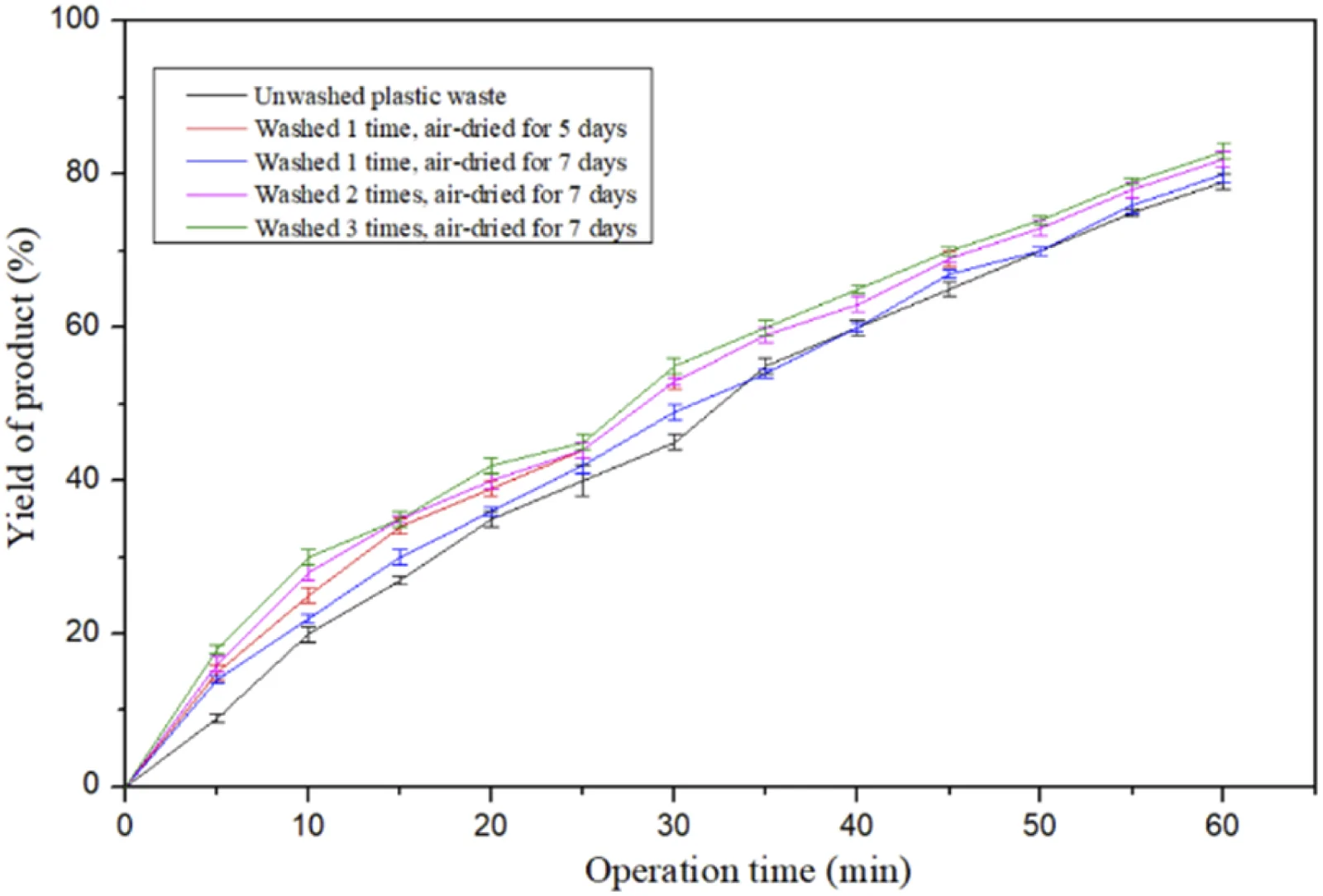
Liquid yield (%) *versus* production time at different numbers of plastic-waste washing cycles.

When these operational parameters were considered synthetically, the optimal condition was identified as a 30-min vacuum duration, 20-kg feed loading, LPG input at ∼25% maximum flow, and a 1-h reaction time at ∼450 °C. Under these conditions, the mean liquid yield reached ∼80 wt% for both washed and unwashed feedstocks (Fig. 5), demonstrating that high liquid recovery is achievable without requiring extensive feedstock pretreatment.

#### 3.1.1 Mass and energy balance analysis

The innovative batch pyrolysis process was operated under an optimized feedstock loading of 20 kg (volumetric capacity ∼1000 L) over a 1-h reaction period. Quantifying the overall mass and energy flows revealed that the thermal energy input provided by LPG combustion was 251.1 MJ (50.22 MJ kg^−1^ × 5 kg LPG). Following thermochemical cracking, the total energy recovered in the derived products reached 768.0 MJ, partitioned as follows:

(1) Solid/gaseous fraction: char and non-condensable gases (4 kg) contributed 72.0 MJ (18.0 MJ kg^−1^ × 4 kg).

(2) Condensed liquid/wax fraction: liquid fuel product (16 kg) yielded 696.0 MJ (43.5 MJ kg^−1^ × 16 kg).

The liquid/wax fraction accounted for 90.6% of the total output energy, demonstrating high energy conversion efficiency and supporting the strong potential of the recovered liquid product as an alternative fuel source.

#### 3.1.2 Performance benchmarking

To validate system performance, key product metrics were quantitatively benchmarked against a related pilot-scale pyrolysis study by Jahirul *et al.* (2023).^31^ Under the optimal conditions established in this work, the system achieved a maximum condensable liquid yield of ∼80 wt%. This recovery rate is highly comparable to the crude oil yield (>80 wt%) reported by Jahirul *et al.*, despite their process requiring a higher thermal severity (540 °C) and a secondary pilot-scale vacuum distillation step (170–380 °C) to recover a refined diesel cut (52.5–53.0 wt%).

Beyond process efficiency, the condensed liquid in this study demonstrated superior environmental and quality profiles. The total sulfur content ranged from 0.0038 to 0.0057 wt%, which is substantially lower than commercial reference fuel oil (1.321 wt%) and typical unrefined pyrolytic fractions. Furthermore, the higher heating value (HHV) reached 43 115–43 500 cal g^−1^ (∼43.1–43.5 MJ kg^−1^), matching the energy density reported for distilled plastic-derived diesel (∼43–44 MJ kg^−1^) by Jahirul *et al.* These findings demonstrate that by optimizing the initial air evacuation strategy (15–20 kPa), high-yield and high-calorific heavy fuel substitutes can be directly produced from contaminated mixed plastic rejects in a single-step batch operation, bypassing the high energy footprint and capital costs associated with post-pyrolysis distillation.

### 3.2. Effect of washing pretreatment and mass/energy balance

Feedstock washing did not yield any meaningful enhancement in liquid recovery. Both washed and unwashed feeds produced equivalent liquid yields of ∼80 wt% under optimized conditions (Fig. 5), confirming that liquid recovery is governed primarily by reactor kinetics and thermodynamics rather than feedstock cleanliness.

To evaluate mass conservation across the 1000-L batch reactor, a complete mass balance was established using the target plastic blend (PP ≥ 45 wt%, LDPE ≥ 45 wt%, PS ≤ 5 wt%, HDPE ≤ 5 wt%). Thermochemical cracking under optimal conditions converted the input feedstock into three distinct streams:

• Liquid product (*Y*_liquid_): dominated the output mass distribution at 70–85 wt%, directly attributed to the high proportion of PP and LDPE, which readily undergo random chain scission into liquid-range hydrocarbons.

• Non-condensable gas (*Y*_gas_): accounted for 15–25 wt%, originating from secondary cracking of volatile intermediates.

• Solid char residue (*Y*_solid_): remained minimal at 0.5–5 wt%, demonstrating complete thermal conversion and negligible inorganic/ash contaminants in the sorted feedstock.

From an energy perspective, processing a standard 20-kg batch required an LPG energy input of 251.1 MJ (based on an LPG calorific value of 50.22 MJ kg^−1^ across 5 kg consumed). The gross energy content embedded in the recovered products totalled 768.0 MJ, comprising 72.0 MJ in the solid/gas stream and 696.0 MJ in the liquid fuel fraction. The liquid fuel fraction contained 90.6% of the total recovered energy, underscoring the exceptional energetic recovery efficiency of this direct vacuum-assisted process.

However, the washing process negatively impacted overall product quality. The water and sediment content of the liquid derived from washed feedstock reached up to 20 vol%, whereas that from unwashed feedstock was only 0.9 vol% (Table 1). This indicates that washing introduces residual moisture or entrains contaminants that persist even after drying. Consequently, evaluating the trade-off between eliminating the washing step to reduce operational expenditure (OPEX) and implementing more intensive drying protocols at higher cost is critical for practical deployment.

**Table 1:** Fuel-relevant properties of the condensed liquid (unwashed *vs.* washed feed) compared with a reference commercial fuel oil

Property	Fuel oil (Cargill Meats (Thailand) Co., Ltd.)	Liquid fuel (unwashed feed, before testing)	Liquid fuel (feed washed 3 cycles, before testing)	Test method
Sulfur (wt%)	1.321	0.0057	0.0038	ASTM D5453-24
Specific gravity at 15.6/15.6 °C	0.9545	0.9327	0.8724	ASTM D4052-22
Viscosity (cSt) at 50 °C	12	12	12	ASTM D445-24
Flash point (°C)	81	62	52	ASTM D93-20
Pour point (°C)	12	23	27	ASTM D97-17b
Heating value (cal g^−1^)	43 619	43 500	43 115	GALLENKAMP auto bomb
Ash (wt%)	0.049	Less than 0.001	Less than 0.001	ASTM D482-19
Water and sediment (vol%)	0.2	0.9	20	ASTM D1796-11
Colour	Black	Black	Black	—
Paraffins (wt%)	12.12	12.57	10.52	ASTM D6730-22
Naphthenes (wt%)	3.71	13.02	9.6	ASTM D6730-22
Olefins (wt%)	12.02	5.45	15.85	ASTM D6730-22
Aromatics (wt%)	15.74	8.59	15.91	ASTM D6730-22
C14+ hydrocarbons (wt%)	56.41	53.45	52.81	ASTM D6730-22

To elucidate this behaviour, moisture content analysis before and after drying was conducted in accordance with ASTM D3173. Moisture content (wt%) was calculated from the initial sample mass (*m*_i_) and the constant dry mass (*m*_d_) *via* eqn.:
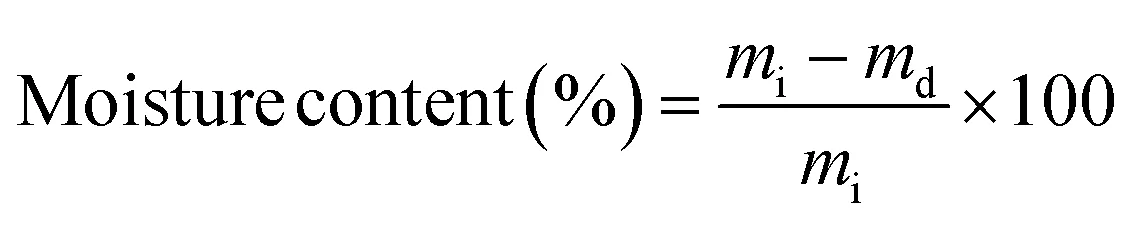


The average moisture contents were 2% for unwashed plastic waste, 18% and 15% for samples washed once and dried for 5 and 7 days, respectively, and 17% for samples washed 2 and 3 times with a fixed 7-day drying period. Insufficient drying left residual surface moisture trapped within the washed plastic flakes, which subsequently vaporized and co-condensed into the liquid fuel product.

### 3.3. Fuel property benchmarking and spectroscopic profiling

The condensed liquid showed several properties comparable to those of commercial fuel oil. Its higher heating value ranged from 43 115 to 43 500 cal g^−1^, close to that of the reference fuel oil (43 619 cal g^−1^), and its viscosity at 50 °C was also comparable, at about 12 cSt, as summarized in Table 1. These results support the fuel-oil-like nature of the product.

As presented in Table 1, the lower flash points (52–62 °C) necessitate the use of explosion-proof equipment and strict adherence to Class II flammable liquid storage standards. Conversely, the higher pour points (23–27 °C) require the installation of heat-traced piping systems for transfer operations under cold climate conditions.

A notable advantage of the pyrolysis liquid was its much lower sulfur and ash content than the reference fuel oil. Sulfur was only 0.0038–0.0057 wt%, and ash was below 0.001 wt%, compared with 1.321 wt% sulfur and 0.049 wt% ash in the reference fuel oil, as summarized in Table 1. These characteristics imply lower potential for SO_*x*_ emissions and ash-related fouling during combustion.

**Fig. 6:**
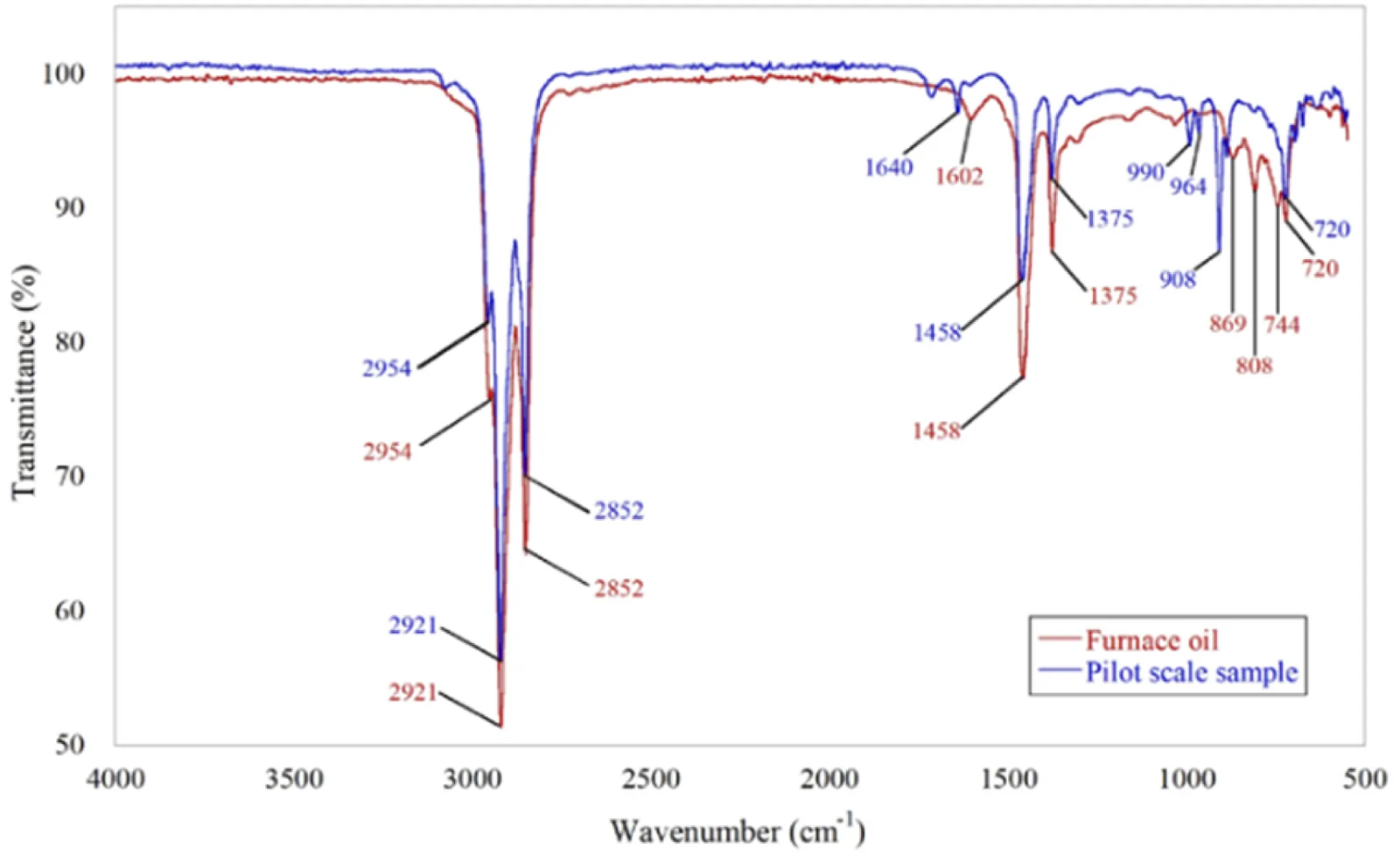
FTIR fingerprints of the pilot scale sample compared with commercial furnace oil.

The FTIR spectra further confirmed the similarity between the condensed liquid and the reference fuel oil, indicating that both were dominated by hydrocarbon-type compounds, as shown in Fig. 6. Together with the reported hydrocarbon distribution, these results suggest that the product can be regarded as a heavy fuel oil-like liquid.

Fig. 6 compares the FTIR spectra of the condensed liquid obtained from the pilot-scale pyrolysis reactor and commercial furnace oil. Both samples exhibit strong, characteristic absorption bands in the aliphatic region (2956, 2920, and 2850 cm^−1^), corresponding to the asymmetric and symmetric C–H stretching vibrations of –CH_3_ and –CH_2_– groups. Additionally, the absorption peaks observed at 1455 cm^−1^ (C–H bending) and 1375 cm^−1^ (C–H symmetric bending) confirm the presence of long-chain aliphatic hydrocarbons (alkanes and alkenes) in both oils.

The pilot-scale sample exhibits prominent absorption peaks at 1640 cm^−1^ (C=C stretching of alkenes) along with out-of-plane bending vibrations at 990, 910, and 890 cm^−1^ (=C–H bending), indicating a notable proportion of unsaturated alkenes produced during thermal cracking. In contrast, the commercial furnace oil displays a distinctive peak at 1600 cm^−1^ (C=C aromatic ring stretching) along with out-of-plane C–H bending vibrations in the range of 700–900 cm^−1^, demonstrating a higher content of aromatic hydrocarbons. The peak at 722 cm^−1^ in both spectra corresponds to the –CH_2_– rocking vibration ((–CH_2_–)_*n*_, *n* ≥ 4), characteristic of straight-chain aliphatic structures. Overall, the FTIR analysis confirms that the chemical structure of the condensed liquid closely resembles that of commercial furnace oil, validating its potential application as a replacement fuel.

FTIR analysis (Fig. 6) demonstrates that the functional groups and chemical structure of the condensed liquid product closely resemble those of commercial heavy fuel oil used in steam generation. Catalytic pyrolysis using zeolites or activated carbon typically aims to maximize light hydrocarbon fractions or aromatics through secondary cracking.^32,33^ However, when processing contaminated municipal plastic rejects, direct exposure to solid catalysts leads to rapid catalyst deactivation, coking, and severe pore plugging caused by inorganic impurities. Furthermore, solid catalysts represent significant operational expenditures (OPEX) and complicate continuous pilot-scale processing. By employing a direct, non-catalytic vacuum-assisted process, we significantly reduce operating costs and process complexity, eliminate catalyst regeneration and disposal requirements, and achieve a high oil yield (∼80 wt%) rich in heavy-to-medium hydrocarbon fractions suitable for direct application as commercial fuel oil substitutes.

GC-MS analysis further confirmed that the condensed liquid closely resembled the reference fuel oil. Representative compounds identified in both samples are listed in Table 1, whereas the hydrocarbon-class distribution is summarized in Table 2. Both fuels were dominated by non-aromatic hydrocarbons, with heavy fractions (>C_13_) accounting for more than 60% of the total composition. The similar carbon-number distribution observed in both samples supports the fuel-oil-like characteristics of the pyrolysis liquid.

**Table 2:** Hydrocarbon distribution of the condensed liquid and commercial fuel oil classified by carbon-number range and hydrocarbon type based on GC-MS analysis

Carbon range	Type	Condensed liquid (% area)	Fuel oil (% area)
C_5_–C_9_	Aromatic	1.59	2.12
	Non-aromatic	14.05	10.72
	Total	15.64	12.84
C_10_–C_13_	Aromatic	7.86	8.15
	Non-aromatic	15.42	14.39
	Total	23.28	22.54
>C_13_	Aromatic	11.57	12.47
	Non-aromatic	51.45	53.26
	Total	63.02	65.73

Table 2 presents the detailed carbon-number distribution and hydrocarbon composition of the condensed liquid compared to commercial fuel oil, as determined by GC-MS analysis. The hydrocarbon profiles of both oils exhibit a high degree of similarity across all carbon number ranges (C_5_–C_9_, C_10_–C_13_, and >C_13_).

Heavy hydrocarbons (>C_13_) constitute the primary fraction in both samples, accounting for 63.02% area in the condensed liquid and 65.73% area in the commercial fuel oil. Non-aromatic hydrocarbons dominate this heavy fraction (51.45% area for condensed liquid *vs.* 53.26% area for fuel oil), which accounts for the high energy density and fuel characteristics comparable to conventional fuel oil. Middle-distillate hydrocarbons (C_10_–C_13_) represent 23.28% area and 22.54% area for the condensed liquid and commercial fuel oil, respectively.

Furthermore, the light fraction (C_5_–C_9_) is slightly higher in the condensed liquid (15.64% area) than in the commercial fuel oil (12.84% area), primarily driven by a higher non-aromatic content (14.05% *vs.* 10.72% area). Across all fractions, the condensed liquid exhibits a marginally lower total aromatic content compared to the commercial fuel oil (C_5_–C_9_: 1.59% *vs.* 2.12%; C_10_–C_13_: 7.86% *vs.* 8.15%; >C_13_: 11.57% *vs.* 12.47%). Overall, the overall aromatic-to-non-aromatic distribution and carbon number ranges strongly indicate that the synthesized condensed liquid possesses fuel characteristics highly compatible with commercial fuel oil, supporting its potential application as a direct fuel substitute without requiring further fractionating steps.

The hydrocarbon distribution observed in this study aligns well with previous findings reported for pilot-scale pyrolysis of mixed polyolefin and real-world plastic wastes. Specifically, the predominance of the >C_13_ fraction (63.02% area) with a high non-aromatic ratio is consistent with the results of Jahirul *et al.* (2023), who demonstrated that thermal cracking of mixed waste plastics naturally favors long-chain aliphatic structures prior to secondary catalytic or vacuum distillation.

Furthermore, the low total aromaticity observed across all carbon ranges (C_5_–C_9_, C_10_–C_13_, and >C_13_) in our direct vacuum-assisted system is in good agreement with the work of Miandad *et al.* (2016)^34^ and Sharuddin *et al.* (2016).^26^ They highlighted that non-catalytic pyrolysis of mixed plastics (such as PE and PP) yields predominantly straight-chain and branched alkanes/alkenes, whereas high aromatic contents are typically formed only when zeolitic catalysts (*e.g.*, HZSM-5) are introduced to promote cyclization and aromatization reactions.

Additionally, the presence of a notable C_10_–C_13_ middle-distillate fraction (23.28% area) alongside the heavy fraction matches the structural trends reported by Siddiqui and Redhwi (2009) for thermal co-pyrolysis of plastic mixtures. This comparative analysis confirms that our single-stage, direct vacuum evacuation process successfully preserves the desired heavy-to-medium hydrocarbon distribution suitable for heavy fuel oil applications, avoiding excessive cracking into light non-condensable gases while suppressing unwanted aromatic by-products.

### 3.4. Operational air quality and environmental safety

Air-quality monitoring was conducted under controlled field conditions, with the prevailing wind direction aligned along the sampling axes and ambient temperatures ranging between 33 and 38 °C. Random atmospheric sampling was executed during the steady liquid-generation phase (0.5–1.0 h post-initiation).

Particulate matter concentrations exhibited pronounced spatial attenuation with increasing distance from the reactor unit. As summarized in Table 3, PM_2.5_ levels decreased from 293 µg m^−3^ at 0 m to 165 µg m^−3^ at 5 m, reaching 22 µg m^−3^ at 10 m. Concurrently, PM_10_ levels dropped from 384 µg m^−3^ at source to 134 µg m^−3^ (5 m) and 28 µg m^−3^ (10 m). Similarly, CO_2_ concentrations declined from 2017 ppm at 0 m to 1097 ppm at 5 m and 500 ppm at 10 m, whereas ambient O_2_ remained stable within 20.0–−20.8%. This profile is consistent with natural atmospheric dispersion rather than localized oxygen depletion, confirming that maintaining a minimum working clearance of 10 m substantially mitigates operator exposure.

**Table 3:** Air quality and exhaust-gas indicators as a function of distance from the product storage tank (0, 5, and 10 m) under the representative operating condition^a^

Air-quality and combustion-efficiency parameters	Position at a distance from the product storage tank		
	0 m	5 m	10 m
HCHO (mg m^−3^)	0.008 ± 0.003	0.007 ± 0.001	0.006 ± 0.002
TVOC (mg m^−3^)	0.015 ± 0.002	0.014 ± 0.002	0.0012 ± 0.0003
PM_2.5_ (µg m^−3^)	293 ± 2.8	165 ± 2.6	22 ± 1.5
PM_10_ (µg m^−3^)	384 ± 2.5	134 ± 2.7	28 ± 1.5
CO (ppm)	1 ± 0.2	0.1 ± 0.05	0 ± 0.0
CO_2_ (ppm)	2017 ± 2.9	1097 ± 3.8	500 ± 4.5
NO_*x*_ (%)	0 ± 0.0	0 ± 0.0	0 ± 0.0
O_2_ (%)	20.0 ± 0.3	20.3 ± 0.5	20.8 ± 0.3

a When 1 ppb = 1 µg L^−1^ = 1 mg m^−3^, and 1 ppm = 1 mg L^−1^ = 1 mg kg^−1^.

To evaluate the broader environmental footprint of the pilot-scale unit, gaseous emissions generated during non-catalytic pyrolysis were contextualized against existing literature. The non-condensable gaseous fraction primarily comprises light hydrocarbons (C_1_–C_4_), CO, and CO_2_, which is characteristic of the thermal cracking of mixed polyolefin rejects, as reported by Munir *et al.* (2021).^33^ Owing to the inherently low sulfur and nitrogen contents of municipal plastic waste, SO_*x*_ and NO_*x*_ emissions remain well below regulatory exposure thresholds, aligning with findings by Miskolczi *et al.* (2004).^27^

Fugitive volatile organic compound (VOC) emissions are effectively suppressed during thermal ramping by integrating a pre-heating vacuum evacuation stage (15–20 kPa). Furthermore, off-gases are routed to a thermal oxidizer coupled with a wet scrubber, utilizing the recovered syngas as auxiliary fuel for the reactor heating jacket, adapted from Jahirul *et al.* (2023).^31^ This dual-mitigation strategy neutralizes trace acid gases, destroys residual VOCs and CO, and enhances overall thermal efficiency and process sustainability for community-scale deployment.

## 4 Conclusions

This study successfully demonstrates the technical feasibility of direct, vacuum-assisted pilot-scale batch pyrolysis for converting mixed non-recyclable plastic residues (PP, LDPE, PS, and HDPE) into high-value fuel oil substitutes. Under optimized operating conditions—comprising a 30-min air pre-evacuation (15–−20 kPa), a 20-kg feed loading, LPG heat input at ∼25% maximum flow, and a 1-h reaction at ∼450 °C—a high condensable liquid yield of ∼80 wt% was achieved without requiring continuous inert-gas purging. Comprehensive characterization confirmed that the pyrolytic liquid is aliphatic-rich and dominated by heavy (>C_13_) and middle-distillate (C_10_–C_12_) fractions with low total aromaticity. The product exhibits superior fuel properties, including a high heating value (43 115–43 500 cal g^−1^; ∼43.1–43.5 MJ kg^−1^) and kinematic viscosity at 50 °C (∼12 cSt) closely matching commercial furnace oil, alongside environmentally advantageous ultra-low sulfur (0.0038–−0.0057 wt%) and ash contents (<0.001 wt%). Furthermore, washing pretreatment provided no benefit in liquid recovery while significantly increasing water and sediment contamination due to trapped residual moisture, confirming that extensive feedstock washing is unnecessary. Spatial air-quality monitoring revealed rapid particulate dispersion (PM_2.5_ and PM_10_ dropped below 28 µg m^−3^ at 10 m), establishing a safe operational buffer distance of ≥10 m. Overall, this single-step, direct vacuum-assisted pyrolysis process offers a cost-effective, energy-efficient, and scalable solution for upcycling post-sorting municipal plastic waste without the need for catalytic upgrading or post-pyrolysis distillation.

## Author contributions

Kanokwan Panmak and Yuvarat Ngernyen: conceptualization, methodology, investigation, formal analysis. Supattra Budsaereechai: conceptualization, methodology, investigation, formal analysis, writing – original draft, supervision, funding acquisition, writing – review & editing.

## Conflicts of interest

There are no conflicts to declare.

## Data Availability

All data generated or analyzed during this study are included in this published article.
